# Natural Variation in Elicitation of Defense-Signaling Associates to Field Resistance Against the Spot Blotch Disease in Bread Wheat (*Triticum aestivum* L.)

**DOI:** 10.3389/fpls.2018.00636

**Published:** 2018-05-16

**Authors:** Sandeep Sharma, Ranabir Sahu, Sudhir Navathe, Vinod K. Mishra, Ramesh Chand, Pawan K. Singh, Arun K. Joshi, Shree P. Pandey

**Affiliations:** ^1^CSIR-Central Salt and Marine Chemicals Research Institute, Bhavnagar, India; ^2^Department of Biological Sciences, Indian Institute of Science Education and Research Kolkata, Kolkata, India; ^3^Institute of Agricultural Sciences, Banaras Hindu University, Varanasi, India; ^4^The International Maize and Wheat Improvement Center (CIMMYT), Texcoco, Mexico; ^5^The International Maize and Wheat Improvement Center (CIMMYT), New Delhi, India

**Keywords:** *Bipolaris sorokiniana*, defense signaling, natural variation, salicylic acid, syringic acid, spot blotch, *Triticum aestivum*, wheat

## Abstract

Spot blotch, caused by the hemibiotropic fungus *Bipolaris sorokiniana*, is amongst the most damaging diseases of wheat. Still, natural variation in expression of biochemical traits that determine field resistance to spot blotch in wheat remain unaddressed. To understand how genotypic variations relate to metabolite profiles of the components of defense-signaling and the plant performance, as well as to discover novel sources of resistance against spot blotch, we have conducted field studies using 968 wheat genotypes at 5 geographical locations in South-Asia in 2 years. 46 genotypes were identified as resistant. Further, in independent confirmatory trials in subsequent 3 years, over 5 geographical locations, we re-characterized 55 genotypes for their resistance (above 46 along with Yangmai#6, a well characterized resistant genotype, and eight susceptible genotypes). We next determined time-dependent spot blotch-induced metabolite profiles of components of defense-signaling as well as levels of enzymatic components of defense pathway (such as salicylic acid (SA), phenolic acids, and redox components), and derived co-variation patterns with respect to resistance in these 55 genotypes. Spot blotch-induced SA accumulation was negatively correlated to disease progression. Amongst phenolic acids, syringic acid was most strongly inversely correlated to disease progression, indicating a defensive function, which was independently confirmed. Thus, exploring natural variation proved extremely useful in determining traits influencing phenotypic plasticity and adaptation to complex environments. Further, by overcoming environmental heterogeneity, our study identifies germplasm and biochemical traits that are deployable for spot blotch resistance in wheat along South-Asia.

## Introduction

Wheat (*Triticum asetivum* L.) is the second most-produced cereal crop, grown on more than 17% of the total cultivable land of the world, with the gross production reaching to 735 million tons after maize (1,027 million tons) in 2015–2016 (http://www.fao.org/worldfoodsituation/csdb/en/; Food and Agricultural Organization of United Nations estimates). Still, wheat production warrants an increase of production by 70% in order to meet the future demands of food security. The challenge to wheat production is further compounded by losses incurred due to wide-spread occurrence of diseases. Spot blotch disease of wheat, caused by *Bipolaris sorokiniana*, has emerged as a critical challenge to wheat cultivation, especially in the warm and humid areas of the world (Dubin and Rajaram, 1996; Nizam et al., 2012; Arseniuk, 2014). *B. sorokiniana* is a hemibiotrophic fungal pathogen (Dubin et al., 1998) that displays a wide variability in natural habitats (Mehta, 1981; Duveiller and Garcia Altamirano, 2000; Chand et al., 2003; Pandey et al., 2008; Asad et al., 2009). Multi-nucleate state, nuclear migration, and heterokaryosis are a few mechanisms giving rise to variability in the pathogen that propagates asexually in warm and humid climates (Chand et al., 2003; Pandey et al., 2008). South-east Asia (Saari, 1998), North and Latin America, Africa (Duczek and Jones-Flory, 1993), India (Joshi et al., 2002), China (Chang and Wu, 1998), and Brazil (Mehta, 1993) are few major affected areas where spot blotch have caused severe reduction in wheat production. On an average, wheat encounters a loss of 17% of yield due to spot blotch but as much as 70% in yield reduction has been reported when plants are infected during grain filling stage whereas, under epidemic conditions, losses may be as high as 100% (Sharma and Duveiller, 2006). Thus, the threat of spot blotch to wheat crop warrants identification of novel genetic sources of resistance. One of the possible ways is to extensively study the molecular basis of wheat-spot blotch interaction to explore the natural variation in the pathogen-induced deployment of components of signal transduction pathways. This would also help to understand the mechanism of resistance against spot blotch pathogen.

Exploring natural variation is highly valuable in uncovering the determinants of phenotypic plasticity and understanding evolution of traits that benefit plant adaptation to rapidly evolving biotic stresses (Li et al., 2015). But few investigations have been undertaken in crop species to study natural variation of metabolic traits (Meihls et al., 2013; Soltis and Kliebenstein, 2015). Study of metabolic variation in natural populations can be highly useful for designing crop improvement programs for disease resistance and yield traits by allowing the assessment of “metabolic network properties” prior to analyses focused on particular loci (Soltis and Kliebenstein, 2015). This can be achieved by evaluating the levels of relevant metabolites in large numbers of natural genotypes when exposed to relevant treatment conditions across environments. Such investigation has not been conducted in wheat for spot blotch infection. Use of host plant resistance is one of the most efficient approaches to control the growth of pathogen and arrest progression of diseases. Natural allelic variation present in existing diverse genotypes provides valuable information about their performances and can be exploited to improve the quantitative traits. There are reports where natural variation within species have been studied and utilized in improving various polygenic traits (Alonso-Blanco et al., 2003, 2009; Meng et al., 2008; Driever et al., 2014), including disease resistance (Kover and Schaal, 2002; Kover et al., 2005; Bomblies et al., 2007). Natural genetic variation for spot blotch resistance has also been reported in wheat (Joshi and Chand, 2002; Joshi et al., 2007; Rosyara et al., 2007, 2009; Gurung et al., 2014; Kumar et al., 2016). However, few or no systematic investigations have been undertaken in agro-ecological habitats to investigate the relationship between natural variation for spot blotch resistance and defense-signaling in wheat.

Recruitment of effective resistance against pathogens involves a complex network of signaling events (Dangl and Jones, 2001; Wiesner-Hanks and Nelson, 2016) that remain poorly studied in wheat-spot blotch interaction. The reactive oxygen species (ROS) play an integral role as signaling molecule in the regulation of defense response, to prevent pathogen infection (Baxter et al., 2014). The rapidly accumulated ROS (at low levels) might act as important signal transduction molecules during early defense response (Kumar et al., 2002; Rodríguez-Decuadro et al., 2014); however, they become toxic if accumulated at higher amounts at later stages of the infection (Camejo et al., 2016). To regulate the toxic levels of ROS and to maintain redox homeostasis in the cells, plants activate anti-oxidant defense machinery involving enzymes such as superoxide dismutase (SOD), catalase (CAT), glutathione peroxidase (GPX), monodehydroascorbate reductase (MDHAR), dehydroascorbate reductase (DHAR), and glutathione reductase (GR) (Sewelam et al., 2016). A close association between high activity of antioxidant enzymes and disease resistance has been reported in a number of plant species (Foyer and Noctor, 2005; Liu et al., 2010; Hakmaoui et al., 2012; Perez and Brown, 2014; Mohapatra et al., 2016), but not in wheat. Similarly, increased levels of phenolic secondary metabolites have been associated with plant resistance against virulent pathogens (Naoumkina et al., 2010; Fang et al., 2013), but a detailed investigation on the natural variation in spot blotch induced secondary metabolites have been limited in wheat.

Change in the redox state during defense response often rely on production of stress specific chemicals including phytohormones (Pieterse et al., 2009; Baxter et al., 2014). Salicylic acid (SA) is a plant signaling hormone, required for both, local defense and systemic acquired resistance (SAR) (Vlot et al., 2009). An increase in the endogenous SA level associates with the resistance of the infected plant to the invading pathogen (Malamy et al., 1990; Métraux et al., 1990; Uknes et al., 1993). An increase in pathogen-induced SA levels result into a massive production of anti-microbial pathogenesis-related (PR) proteins, thus SA may promote plant immunity against a broad spectrum of pathogens through the combined activities of these anti-microbial proteins (Wang et al., 2005). We have recently shown that SA levels increase in resistance response to spot blotch infection (Sahu et al., 2016a,b). However, natural variation for spot blotch induced SA accumulation in wheat have not yet been studied.

The aim of present study was to exploit natural variation to identify novel sources of spot blotch resistance in wheat and to assess the genotypic variation in defense signaling that may be responsible for inducing effective resistance in agro-ecological habitats, providing the possibilities of their use in wheat breeding to enhance disease resistance.

## Materials and methods

### Plant material and growth conditions

**Figure 1:**
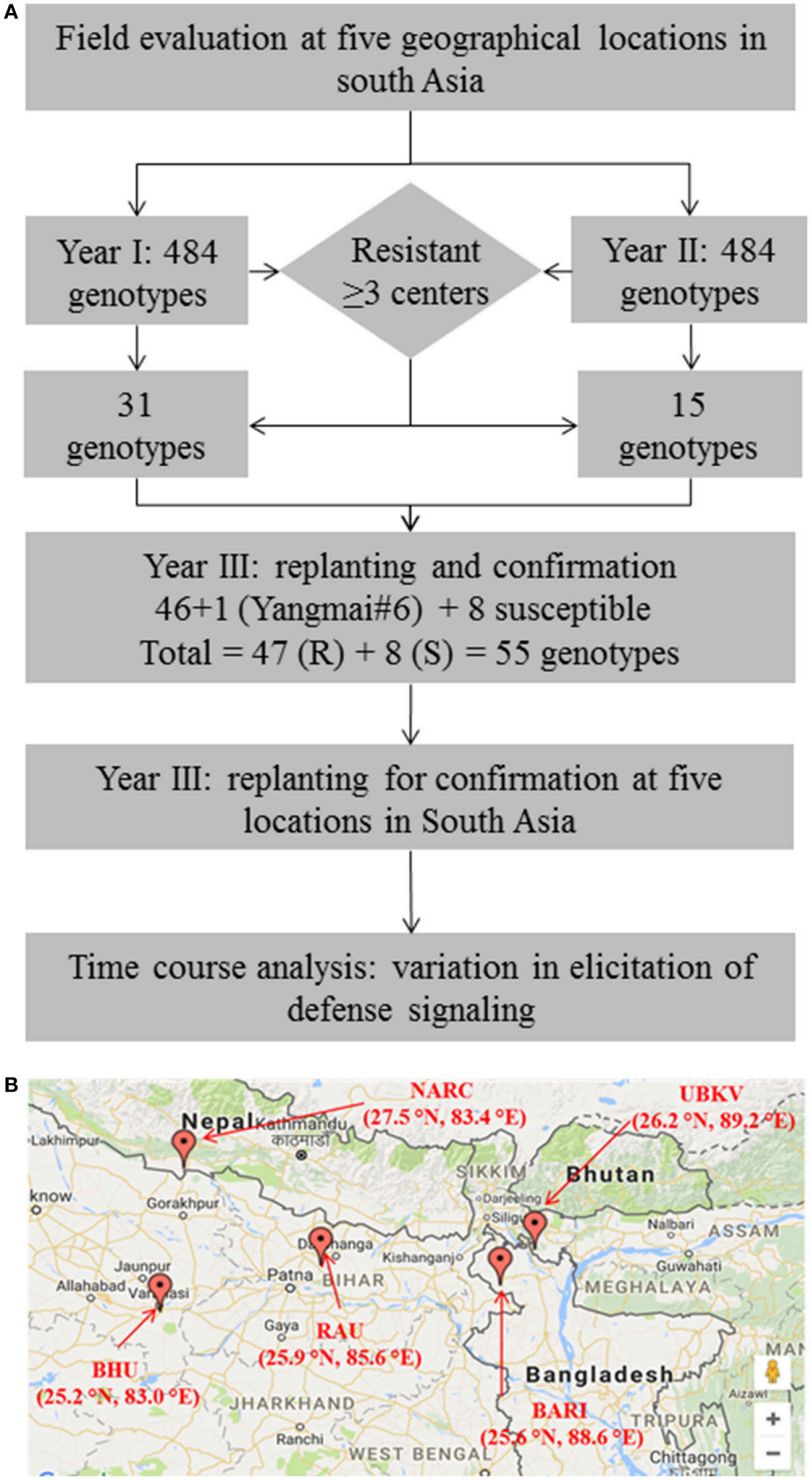
Evaluation of wheat germplasm in South-Asia identifies promising sources of resistance against spot blotch. **(A)** Schematic summarizes the experimental design adapted in the present study. **(B)** A geographical map showing the locations across South-Asia (India, Nepal and Bangladesh) where field experiments were conducted. Thirty-one genotypes in the year 1, and 15 in year 2 trials were identified as resistant.

A total of 968 wheat genotypes (CIMMYT and national wheat improvement program; Supplementary Table 1) were compiled (referred to here as CRP genotypes as they are the part of CGIAR Research Program on Wheat funded project) and screened in 2 years in sets of 484 genotypes each in the years 2012-13 and 2013-2014, at five independent geographical locations in South-Asia as detailed below (Figures 1A,B). All the plant material collected and used in this study is summarized in Table 1A. The first set of germplasm of 484 genotypes (screened in 2012–13; Supplementary Table 1A) consisted of the WAM, 3rd CSISA SB, CIMCOG and 44th IBWSN populations (detailed description in Table 1A). The second set of 484 genotypes (Supplementary Table 1B; screened in 2013-14), were from 4th CSISA-SB (Singh et al., 2015), 4th CSISA HT-EM, 45th IBWSN, 33rd ESWYT, 2nd SATYN, 20th SAWYT, and 1st WYCYT populations, as detailed in Table 1A. All the genotypes were screened at 3 sites in India: Banaras Hindu University (BHU; 25.2 °N, 83.0 °E), Rajendra Agriculture University (RAU; 25.2 °N, 83.0 °E), and Uttar Banga Krishi Viswavidyalaya (UBKV; 26.2 °N, 89.2 °E), as well as at the Wheat Research Centre (WRC) of Bangladesh Agriculture Research Institute (BARI, Dinajpur, Bangladesh; 27.5 °N, 83.44 °E) and National Wheat Research Program (NWPR) of Nepal Agriculture Research Council (NARC, Bhairahawa, Nepal; 27.5 °N, 83.4 °E) in alpha-lattice design, where each genotype was planted in two rows of 2 m each. Alpha-lattice design was generated using PROC PLAN method in SAS software v9.2. α(0, 1) lattice method was used as blocking criterion. In total, 22 blocks, having 22 genotypes in each block, thus comprising 484 genotypes in each replication, were planted. Row-to-row and plant-to-plant distance was 25 and 5 cm, respectively. The whole setup was independently duplicated at all the five sites. Fertilizers (nitrogen (N), phosphorus (P) and potassium (K) in a ratio of 120:60:40 were applied according to standard agronomic practice.

Table 1Evaluation of wheat genotypes for determining sources of resistance against spot blotch disease in South-Asia.

**Table:** 

**(A) Plant material and populations used in the present study to screen genotypes resistant to spot blotch disease in field conditions of South-Asia**.		
**Name of the population**	**Genotype numbers**	**Screening year**
Wheat association mapping (WAM)	294	2012–13
3rd cereal system initiative in south Asia spot blotch (3rd CSISA SB)	52	2012–13
CIMMYT core germplasm (CIMCOG)	60	2012–13
44 international bread wheat screening nursery (44 IBWSN)	78	2012–13
4 cereal system initiative in south Asia-spot blotch (4 CSISA-SB)	50	2013–14
4 cereal system initiative in south Asia heat-early maturity (4 CSISA HT-EM)	29	2013–14
45 international bread wheat screening nursery (45 IBWSN)	251	2013–14
33 elite selection wheat yield trials (33 ESWYT)	50	2013–14
2 stress adaptive trait yield nursery (2 SATYN)	43	2013–14
20 semi-arid wheat yield trial (20 SAWYT)	42	2013–14
1 wheat yield consortium yield trail (1 WYCYT)	19	2013–14

**Table:** 

**(B) Evaluation of wheat genotypes for determining sources of resistance against spot blotch disease in South-Asia; Pedigree of genotypes selected from the field screening (2012–14) and used in confirmatory trials (2014–2017)**.		
**Pedigree**	**Entry numbers (2012–14)**	**Entry/CRP Number (2014–17)**
**GENOTYPES SELECTED FROM SCREENING IN 2012-13**		
CROC_1/AE.SQUARROSA (205)//KAUZ/3/ENEIDA	192	CRP2
ASTREB/OAX93.10.1//SOKOLL	335	CRP3
CHIRYA.3	337	CRP4
SURUTU-CIAT	89	CRP6
CNDO/R143//ENTE/MEXI_2/3/AEGILOPS SQUARROSA (TAUS)/4/WEAVER/5/PASTOR	228	CRP7
MILAN/KAUZ/3/URES/JUN//KAUZ/4/CROC_1/AE.SQUARROSA (224)//OPATA	236	CRP8
YAV_3/SCO//JO69/CRA/3/YAV79/4/AE.SQUARROSA (498)/5/2*OPATA	294	CRP9
TILHI/SOKOLL	297	CRP10
BCN/RIALTO	346	CRP11
UP2338*2/4/SNI/TRAP#1/3/KAUZ*2/TRAP//KAUZ/5/MILAN/KAUZ//CHIL/CHUM18/6/UP2338*2/4/SNI/TRAP#1/3/KAUZ*2/TRAP//KAUZ	390	CRP12
PBW 343/PASTOR	448	CRP14
TILHI	48	CRP15
NL 750	65	CRP16
SW89-5124*2/FASAN	68	CRP17
W462//VEE/KOEL/3/PEG//MRL/BUC	79	CRP18
CNDO/R143//ENTE/MEXI_2/3/AEGILOPS SQUARROSA (TAUS)/4/WEAVER/5/2*KAUZ	93	CRP19
JUPARE C 2001	98	CRP20
ATTILA/3*BCN//BAV92/3/TILHI	99	CRP21
ALTAR 84/AEGILOPS SQUARROSA (TAUS)//OPATA	153	CRP22
ALTAR84/AE.SQUARROSA (219)//OPATA/3/WBLL1/FRET2//PASTOR	291	CRP23
GAN/AE.SQUARROSA (897)//OPATA/3/BERKUT	293	CRP25
ATTILA/3*BCN//BAV92/3/TILHI/4/SHA7/VEE#5//ARIV92	301	CRP26
VORB/4/D67.2/PARANA 66.270//AE.SQUARROSA (320)/3/CUNNINGHAM	321	CRP27
ASTREB/OAX93.10.1//SOKOLL	336	CRP28
CMH79A.955/4/AGA/3/4*SN64/CNO67//INIA66/5/NAC/6/RIALTO	356	CRP29
CROC_1/AE.SQUARROSA (205)//KAUZ/3/SASIA/4/TROST	406	CRP30
BECARD	417	CRP32
PFAU/MILAN//TROST/3/PBW65/2*SERI.1B	419	CRP33
TILHI/PALMERIN F2004	426	CRP34
PBW343*2/KUKUNA//PBW343*2/TUKURU/3/PBW343	432	CRP35
NL748/NL837	443	CRP36
**GENOTYPES SELECTED FROM SCREENING IN 2013-14**		
PBW343*2/KUKUNA//TECUE #1	364.0	CRP38
UP2338*2/KKTS*2//YANAC	473.0	CRP40
ATTILA*2/PBW65*2//MURGA	384.0	CRP41
UP2338*2/KKTS*2//YANAC	229.0	CRP42
MURGA/KRONSTAD F2004	463.0	CRP43
SAUAL/KIRITATI//SAUAL	214.0	CRP44
BAV92//IRENA/KAUZ/3/HUITES/4/FN/2*PASTOR/5/BAV92//IRENA/KAUZ/3/HUITES	272.0	CRP45
BABAX/LR39//BABAX/3/VORB/4/SUNCO/2*PASTOR	442.0	CRP46
ATTILA*2/PBW65*2//MURGA	244.0	CRP47
KACHU #1/4/CROC_1/AE.SQUARROSA (205)//KAUZ/3/SASIA/5/KACHU	457.0	CRP49
SKAUZ*2/FCT'S'//VORB	471.0	CRP50
OPATA//SORA/AE.SQUARROSA (323)	94.0	CRP51
CNDO/R143//ENTE/MEXI_2/3/AEGILOPS SQUARROSA (TAUS)/4/WEAVER/5/PICUS/6/2*PBW65/2*PASTOR	451.0	CRP52
SAUAL/KIRITATI//SAUAL	213.0	CRP52
BABAX/KS93U76//BABAX/3/2*SOKOLL	40.0	CRP54
**CONTROL RESISTANT (R) AND SUSCEPTIBLE (S) GENOTYPES / CHECKS**		
SONALIKA	398 (S)	CRP1
W15.92/4/PASTOR//HXL7573/2*BAU/3/WBLL1	23 (S)	CRP5
TILILA/TUKURU/4/SERI.1B*2/3/KAUZ*2/BOW//KAUZ	230 (S)	CRP13
CIANO T 79	435 (S)	CRP24
MEX94.2.19	104 (S)	CRP31
PFAU/WEAVER*2/4/BOW/NKT//CBRD/3/CBRD	276 (S)	CRP39
WBLL1*2/KURUKU*2/5/REH/HARE//2*BCN/3/CROC_1/AE.SQUARROSA (213)//PGO/4/HUITES	249 (S)	CRP48
WBLL1*2/KUKUNA//AKURI #1	343 (S)	CRP55
Yangmai#6	Resistant	CRP37

**Table:** 

**(C) Evaluation of wheat genotypes for determining sources of resistance against spot blotch disease in South-Asia; Performance of 55 genotypes of wheat (Table** **1B****), during 2014–17 (confirmatory analysis), at all five geographical locations**.											
		**Area Under Disease Progress Curve (AUDPC)**									
		**BHU**		**BARI**		**UBKV**		**RAU**		**NWPR**	
**S. No**.	**Disease response**	**AVG**	**SD**	**AVG**	**SD**	**AVG**	**SD**	**AVG**	**SD**	**AVG**	**SD**
**CRP1**	**Susceptible**	**721.6**	**56.4**	**705.6**	**74.4**	**721.0**	**119.4**	**757.4**	**70.3**	**635.3**	**30.8**
CRP2	Resistant	315.3	26.3	288.0	18.1	169.9	13.3	298.3	34.3	258.9	17.7
CRP3	Resistant	287.9	33.3	326.2	52.6	287.1	34.3	328.7	42.7	336.0	15.5
CRP4	Resistant	236.2	16.2	253.5	32.0	232.5	45.2	262.9	40.9	206.7	20.4
**CRP5**	**Susceptible**	**700.0**	**42.2**	**524.1**	**23.8**	**540.4**	**39.2**	**621.0**	**47.5**	**653.5**	**37.9**
CRP6	Resistant	141.8	4.0	311.9	32.0	206.7	31.7	255.2	45.2	221.6	27.6
CRP7	Resistant	187.3	23.4	285.7	31.5	242.3	39.1	262.0	27.9	235.0	29.9
CRP8	Resistant	252.5	13.8	310.5	26.4	269.2	20.9	301.0	22.1	276.0	28.1
CRP9	Resistant	174.0	26.6	227.9	26.9	213.1	50.4	288.8	23.1	209.1	22.8
CRP10	Resistant	258.0	6.8	291.8	35.6	264.3	46.2	280.6	20.9	262.9	27.9
CRP11	Resistant	236.0	8.5	306.6	31.5	300.8	33.5	208.0	35.5	295.2	6.7
CRP12	Resistant	279.3	27.5	287.7	33.9	293.7	31.1	258.3	34.6	286.7	19.5
**CRP13**	**Susceptible**	**687.5**	**45.0**	**629.9**	**39.0**	**749.9**	**97.1**	**658.6**	**34.1**	**621.2**	**30.6**
CRP14	Resistant	356.0	23.3	306.0	19.1	158.8	18.2	310.6	22.4	344.6	17.7
CRP15	Resistant	311.4	5.7	308.8	25.2	282.2	30.0	323.5	29.7	322.3	13.0
CRP16	Resistant	167.7	21.9	202.5	39.1	199.8	32.1	273.6	47.0	262.0	20.1
CRP17	Resistant	285.9	24.9	328.2	32.6	210.7	21.9	278.5	32.9	289.5	14.2
CRP18	Resistant	298.8	15.8	296.6	40.7	266.3	18.1	305.1	22.1	288.6	25.1
CRP19	Resistant	188.0	6.9	239.8	27.2	192.6	33.8	232.6	26.1	251.2	24.9
CRP20	Resistant	331.6	20.8	328.5	20.1	198.7	18.6	293.1	26.2	246.8	23.4
CRP21	Resistant	292.7	23.2	275.8	34.5	182.8	35.2	284.7	29.0	280.7	24.4
CRP22	Resistant	333.6	22.6	281.9	27.1	213.2	14.5	237.7	28.3	242.2	44.7
CRP23	Resistant	289.5	19.3	319.3	22.1	191.3	52.3	333.7	26.4	250.5	25.3
**CRP24**	**Susceptible**	**621.1**	**30.6**	**730.7**	**62.6**	**714.8**	**19.5**	**701.3**	**39.9**	**768.8**	**55.5**
CRP25	Resistant	266.3	38.5	242.6	37.6	288.8	23.5	212.5	24.9	194.2	20.2
CRP26	Resistant	160.6	12.2	318.5	25.1	203.3	39.7	279.4	57.0	281.6	16.1
CRP27	Resistant	205.2	17.9	299.7	31.5	249.8	33.9	286.3	28.5	268.7	20.8
CRP28	Resistant	319.8	21.2	283.8	41.2	201.8	19.0	320.0	22.7	363.8	10.8
CRP29	Resistant	194.0	21.3	319.2	7.9	233.3	38.9	278.2	27.1	287.0	29.8
CRP30	Resistant	328.9	13.8	279.2	30.4	237.0	27.5	355.4	15.8	331.5	15.8
**CRP31**	**Susceptible**	**732.9**	**16.6**	**699.4**	**83.1**	**861.0**	**64.5**	**645.7**	**43.2**	**639.4**	**48.6**
CRP32	Resistant	244.1	13.3	258.8	18.8	211.4	15.8	263.7	23.2	350.9	23.3
CRP33	Resistant	190.8	16.3	338.4	32.7	247.1	16.2	191.7	24.2	280.4	25.4
CRP34	Resistant	241.7	24.4	280.7	35.5	137.9	19.8	262.8	39.7	230.9	22.4
CRP35	Resistant	157.0	12.1	301.9	29.4	134.6	16.9	250.4	40.9	227.5	32.9
CRP36	Resistant	268.9	32.1	271.7	26.5	173.8	20.8	275.0	26.5	277.7	19.9
CRP37	Resistant	218.9	41.7	256.8	42.9	191.9	12.4	241.3	29.9	235.5	19.5
CRP38	Resistant	147.4	12.6	221.6	23.1	164.2	14.9	264.1	33.3	201.7	34.3
**CRP39**	**Susceptible**	**363.0**	**31.5**	**537.9**	**50.4**	**670.3**	**38.6**	**853.9**	**49.3**	**629.1**	**17.3**
CRP40	Resistant	224.7	20.8	242.4	35.5	248.4	18.6	252.9	45.6	244.9	30.4
CRP41	Resistant	278.6	35.9	240.9	29.4	228.0	13.0	248.5	44.3	213.6	22.6
CRP42	Resistant	198.8	3.9	261.8	30.7	249.4	30.7	235.6	44.6	234.8	13.8
CRP43	Resistant	216.3	11.4	247.9	33.0	228.0	28.0	240.4	33.7	257.5	35.1
CRP44	Resistant	209.7	24.3	275.8	21.9	236.9	17.1	258.7	34.8	239.5	15.4
CRP45	Resistant	245.7	14.6	276.3	31.7	174.5	16.2	261.1	31.8	256.9	14.6
CRP46	Resistant	203.7	22.9	289.6	24.9	258.1	9.2	270.0	18.6	281.2	19.5
CRP47	Resistant	255.7	9.8	269.5	24.6	178.5	19.7	269.9	49.0	284.7	27.6
**CRP48**	**Susceptible**	**610.7**	**8.6**	**533.8**	**44.3**	**676.7**	**50.5**	**746.6**	**21.0**	**609.0**	**17.8**
CRP49	Resistant	224.7	8.0	302.6	29.7	218.6	25.2	262.0	39.7	214.6	17.5
CRP50	Resistant	207.6	21.8	249.0	37.6	228.0	37.4	280.9	43.0	254.0	22.2
CRP51	Resistant	247.0	11.9	240.8	34.6	202.9	44.7	278.0	31.4	258.6	14.9
CRP52	Resistant	192.1	7.4	307.5	40.5	202.1	5.5	275.2	19.4	336.6	30.8
CRP53	Resistant	164.3	12.8	250.8	30.2	230.7	32.3	353.2	8.0	256.1	23.7
CRP54	Resistant	369.5	27.5	342.9	48.1	213.4	12.0	303.9	25.0	329.8	8.8
**CRP55**	**Susceptible**	**555.4**	**14.6**	**697.7**	**59.7**	**814.3**	**68.7**	**720.5**	**22.2**	**686.7**	**21.6**
	MSD (0.05)	74.5		78.3		89.3		110.3		102.0	

*Control genotypes (8 susceptible and 1 resistant) were also planted for comparisons*.

*Further grouping of these genotypes separated susceptible genotypes (CRP1, CRP5, CRP13, CRP24, CRP31, CRP39, CRP48, and CRP55) from the resistant on the basis of AUDPC values as represented in table. Minimum significant difference (MSD) was calculated to show the significant difference between resistant and susceptible genotypes at p < 0.05. Susceptible genotypes are shown in the bold letters*.

We used a stringent, two-pronged criterion for selection of resistant genotypes by analyzing the area under disease progress curve (AUDPC, detailed below; Supplementary Table 2). First, a cutoff [equal to lowest AUDPC + LSD (least significant difference)] was used to review the genotype performance in terms of resistance to spot blotch pathogen at a given location. All the genotypes having AUDPC of less than the cutoff were selected as a primarily resistant group of genotypes at a given location. Next, such groups of genotypes across all the five locations were compared. Only the genotypes that were “resistant” in at least 3 of 5 locations were selected for further investigations. Same exercise was conducted in both the years, thus a total of 31 genotypes were selected in year 1, and 15 were selected in year 2 (Supplementary Table 2). Additionally, a principal component analysis (PCA) of performance of 484 genotypes in each year was also conducted (Supplementary Figure 1).

“Resistant” nature of these selected genotypes (a total of 46 genotypes) was reconfirmed by independent screenings for three consecutive years (2014–2017) in field conditions at all the five locations (Figure 1). Yangmai #6, which is proven as a resistant genotype (Sahu et al., 2016b), was also included in the confirmatory trial, making the total number of resistant genotypes as 47. Also, eight susceptible genotypes were included as controls or reference set for comparison of traits in the confirmatory trials. Thus, for confirmatory analysis, a total of 55 lines were evaluated in randomized block design with three replicates where 2 m single row was assigned for each line. Row-to-row and plant-to-plant distance was 25 and 5 cm, respectively. These 55 genotypes were also used in biochemical characterizations.

### Inoculation of pathogen

A pure culture of *B. sorokiniana* (strain HD-3069) was maintained on PDA (potato dextrose agar) media (Chand et al., 2003; Pandey et al., 2008). Sorghum grains were used for large-scale multiplication of spores of *B. sorokiniana* (Chand et al., 2013). For creating artificial epiphytotic in wheat lines, spores suspension was inoculated by spraying at a concentration of 10^4^ spore ml^−1^ of water at the growth stage (GS) 55 (Zadoks et al., 1974) during evening time. After inoculation, a light irrigation was given to maintain a conducive environment for disease development.

### Assessment of disease

Disease was scored at three different growth stages of wheat (Zadoks et al., 1974), GS 63 (beginning of anthesis to half complete), GS 69 (anthesis complete) and GS 77 (late milking), using double digit (DD, 00–99) scale (Saari and Prescott, 1975). The first digit (D1) indicates vertical disease progress on the plant, and the second digit (D2) indicates severity, measured in diseased leaf area. The DS percentage for each score was based on the following formula:

$$\text{\% severity}=(\text{D}1/9){\;}^{*}\;(\text{D}2/9){\;}^{*}\;100$$

Disease progression or AUDPC was calculated using the percent severity estimations corresponding to the disease rating (Shaner and Finney, 1977; Madden et al., 2007) as:

$$\text{AUDPC}=\sum_{\text{i}=1}^{\text{n}}\left[\left\{\left({\text{Y}}_{\text{i}}+{\text{Y}}_{\text{i}+1}\right)/\;2\right\}{\;}^{*}\;\left({\text{t}}_{\text{i}+1}-{\text{t}}_{\text{i}}\right)\right]$$

Where Y_i_ = disease level at time t_i_

(t_i+1_ − t_i_) = days between two disease scores

n = number of readings.

### Associated fitness parameters

In addition to disease severity, days to heading (DH), thousand kernel weight (TKW) and plot yield (amount of harvested grain per crop area) were also recorded to evaluate plant performance.

### Sampling for experiment

Time course experiments were conducted to explore the elicitation of H_2_O_2_, SOD, MDA, SA, and phenolic acids (which may also act as phytoalexins) during the spot blotch progression. Flag-leaves were collected after inoculation of *B. sorokiniana*. Three-five biological replicates at 0, 12, 24, 48, and 72 h post inoculation (hpi) were harvested in liquid nitrogen and stored in −80°C until further use. Samples at 0 hpi (just at the time of inoculation) served as the control/references.

### Estimation of H_2_O_2_

H_2_O_2_ was estimated by modified protocol of Patterson et al. (1984). Leaf samples (200 mg) were homogenized in 2 ml of sodium-phosphate buffer (pH 6.5). The extract was centrifuged at 10,000 rpm for 15 min. Reaction was initiated by adding 1 ml of TiSO_4_ (HiMedia, GRM2484) (1% in 20% H_2_SO_4_) to supernatant. Reaction mixture was centrifuged at 6,000 rpm for 15 min. Absorbance was recorded at 410 nm on double beam UV-VIS spectrophotometer (UV-1800, Shimadzu). The quantity of H_2_O_2_ was expressed as unit per gram fresh weight using extinction coefficient 53.5714.

### SA and phenolics estimation

SA was evaluated as previously described (Sahu et al., 2016a,b). Briefly, 150–200 mg of flag leaves were pulverized with liquid nitrogen and extracted with 1 ml of 90% methanol followed by centrifuged at 13,200 rpm for 15 min at 4°C. Supernatant was recovered and pellet was re-extracted with 1 ml of absolute methanol and supernatants were pooled and dried. 5% trichloroacetic acid was added to the dried samples, followed by addition of 800 μL extraction buffer (ethyl acetate:cyclohexane, 1:1). Upper organic layer was separated and dried for SA estimation by adding UPLC eluent and transferred to a clean vial for analysis.

For total phenolic content and UPLC-based analysis, phenolic acids were extracted as described earlier (Sahu et al., 2016b) in both free and bound form. Flag leaf samples (100–150 mg) were pulverized with liquid N_2_ and extracted with 1 ml of 70% methanol. After centrifugation, the process was repeated and supernatants were combined and used for free phenolic acid contents where pellets were analyzed for bound phenolic acid contents after hydrolysis. Total phenolic contents were determined with the Folin-Ciocalteu's assay (Kofalvi and Nassuth, 1995; Pandey and Baldwin, 2008). Total phenolics content was expressed as μg gallic acid equivalents (GAE). Estimation of individual phenolic acids (4-hydroxybenzoic acid, syringic acid, vanillic acid, *p*-coumaric acid, chlorogenic acid, caffeic acid, sinapic acid and ferulic acid) was investigated using UPLC-DAD method using C18 column (Sahu et al., 2016b). Water and acetonitrile (80:20 v/v) with 0.1% formic acid (pH 3.0) was used as mobile phase at the flow rate of 0.3 ml min^−1^ where column was maintained at 40°C. DAD detector was set with four wavelengths (275, 285, 310, and 320 nm) for UPLC analysis.

### Estimation of SOD

SOD was estimated as described earlier (Dhindsa et al., 1981; Xu et al., 2013). 100 mg leaf samples were crushed in 5 ml of extraction buffer (0.1 M phosphate buffer), centrifuged at 9,000 rpm for 15 min and supernatant (source of enzyme) was collected. Three ml of the reaction mixture containing 0.1 ml of 1.5 M sodium carbonate, 0.2 ml of 200 mM methionine, 0.1 ml of 2.25 mM NBT, 0.1 ml of 3 mM EDTA, 1.5 ml of 100 mM potassium phosphate buffer, 1 ml of distilled water and 0.1 ml of enzyme extract. Two test tubes without enzyme extracts were taken as control. The reaction was started by adding 0.1 ml of riboflavin (60 μM) and placing the test tubes below a light source of two tungsten incandescent light bulb having illuminance of 8072.93 lux for 15 min. A non-irradiated complete mixture that did not develop color served as a blank. Absorbance was recorded at 560 nm in spectrophotometer. The enzyme unit (EU) was expressed on per gram fresh weight basis.

### Estimation of MDA

Lipid peroxidation was estimated as the MDA content, determined by the method of Heath and Packer (1968). Plant samples (100 mg) were homogenized in 5 ml of 0.1% (w/v) of trichloro-acetic acid solution. The homogenate was centrifuged at 9,000 rpm for 20 min and 0.5 ml supernatant was added to 1 ml of 0.5% (w/v) thiobarbituric acid in 20% TCA. The mixture was heated 95°C for 20 min in a water bath and immediately cooled on ice. The samples were again centrifuged at 9,000 rpm for 5 min. The absorbance was recorded at 532 and 600 nm. The amount of MDA-TBA complex (red pigment) was calculated from the extinction coefficient as 155 mM^−1^ cm^−1^. Results are presented as μmoles MDA g^−1^ fresh weight.

### Antifungal activity of syringic acid

PDA plates containing phenolic acids in a dilution series in the concentrations of 0–1,000 μg/ml of syringic acid were prepared and *B. sorokiniana* was allowed to grow on these plates. Diameter (cm) of growth was recorded upto 10 day to determine inhibition of growth. Cultures growing on PDA only (0 μg/ml of phenolic acids) were used as controls for comparison.

### Exogenous complementation assay of syringic acid to determine its effect on disease progression

Role of syringic acid in plant defense was validated by exogenously complementing the spot blotch susceptible Sonalika genotype with syringic acid and determining the gain in resistance (or reduction in susceptibility). Sonalika plants were treated with a foliar spray of syringic acid (TCI Chemicals, India; product number, G0014) at concentrations of 0.1, 0.2, 0.5 mM until run-off. Syringic acid was prepared in 100% methanol and diluted in double distilled water. For comparisons, Sonalika plants (control, 0 mM) were sprayed with only double distilled water containing equal amount of methanol as required to dissolve syringic acid. Twenty four hours after spraying syringic acid, the plants were inoculated with *B. sorokiniana* spore suspension (foliar spray as described above, at concentrations of 10^6^ spores/ml until run-off). The disease severity (DS) (%) was scored on 5, 7, 9 days post inoculation (dpi). Six-seven replicates were used for each condition. The plants were scored on the basis of the Saari and Prescott double digit scale (DD, 00–99), disease severity (DS; %) was scored, and AUDPC was calculated as described above. Plants were grown under controlled conditions at a photoperiod of 14(25°C)/8(22°C) at a relative humidity of 73-75%. Fertilizers (NPK, at a proportion of 19:19:19) were supplied at 15 and 45 days of germination.

### Statistical analysis

Analysis of lattice design was done using PROC MIXED model in SAS v9.2. Analysis of variance (ANOVA) was performed in general linear model (GLM). Dunnett's test; *p* ≤ 0.05 and Tukey-Kramer test; *p* ≤ 0.05 was used to detect significant differences between each genotype under different treatments. Data was expressed as the mean ± standard deviation. R statistical package was also used for PCA analysis. Analysis of correlation was performed by calculating Pearson correlation coefficient (r) between the highest levels of accumulated metabolite and the AUDPC, similar to one described earlier (Sahu et al., 2016b). SA, H_2_O_2_, vanilic acid, chlorogenic acid, p-caumaric acid, and free ferrulic acid were evaluated for correlation analysis at 12 hpi, total phenolic content, 4-hydroxybenzoic acid, syringic acid, caffic acid and bound ferrulic acid were evaluated at 24 hpi, whereas SOD and MDA contents were evaluated at 72 hpi (further detailed in results).

## Results

### Natural variation for spot blotch resistance in wheat

A total of 968 wheat genotypes (Supplementary Table 1) were screened at five geographical locations in 2 years to evaluate the variability for spot blotch resistance (Figure 1). Disease progression (in form of AUDPC) was calculated and genotypes were categorized into resistant and susceptible (detailed in Methods sections). From the field studies of year 1 (2012–13), 31 genotypes were found resistant (Supplementary Figure 1 and Supplementary Table 2): Three genotypes (192, 335, and 337) were resistant at all the five locations, 8 genotypes (89, 228, 236, 294, 297, 346, 390, and 483) were resistant at four locations, whereas 20 were screened as resistant at the three locations. All the resistant genotypes showed large variation for plot yield (149.1–283.1 g/plant), days to heading (75.7–87.7) and TKW (24.5–41.7 g) (Supplementary Table 1). Similarly, 15 wheat genotypes were screened as “resistant” from the second year (2013–14) of field studies (Supplementary Figure 1 and Supplementary Table 2). PCA clusters also largely coincided with the phenotypic selection (Supplementary Figure 1).

A total of 46 wheat genotypes were finally identified as spot blotch resistant from the field screening (Supplementary Table 1). All these 46 genotypes were confirmed as “resistant” in subsequently independent field studies at all the five locations over 3 years (2014–17; Figure 1 and Table 1C and Supplementary Table 3). Pedigree (along with genotype identifiers of field trails the entry and CRP numbers) of these 46 genotypes, a previously characterized resistant genotype (check), Yangmai #6, and eight susceptible controls are given in Table 1B. Hereafter, these genotypes are referred as CRP1-55. The mean AUDPC varied from 134.6 to 369.5 for resistant genotypes, including the 47th resistant genotype (Yangmai #6). As expected, on the other hand, AUDPC values of most of the susceptible genotypes were much higher and varied in the range of 524.1–861.0 (with an exception of genotype CRP39 at BHU Varanasi location with an AUDPC of 363) (Table 1C). The mean variation in plot yield for resistant (150.6-411 g/plant) and susceptible (143.8–374.3 g/plant) genotypes were also measured (Supplementary Table 4). A comparative analysis of 55 genotypes on the basis of AUDPC separated the susceptible and resistant genotypes and ranked them differently for disease progression response (Supplementary Figure 2, Supplementary Table 3). These observations suggest that the 46 genotypes identified in field trails could be used as sources of resistance against spot blotch across South-Asia.

### Spot blotch infection alters redox balance in wheat genotypes

**Figure 2:**
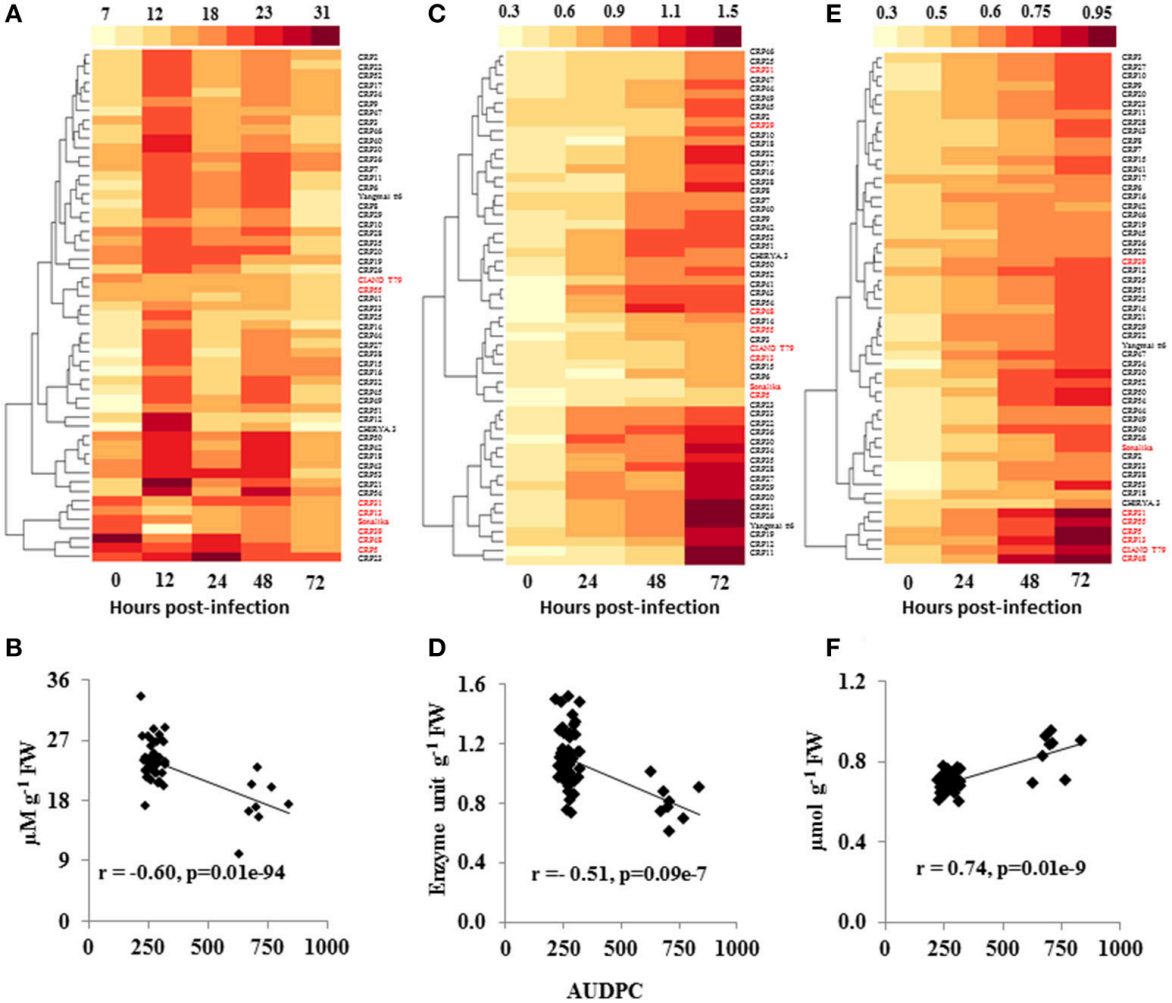
Variation in ROS signaling during spot blotch infection of wheat germplasm. Heatmaps show the H_2_O_2_ levels (μM g^−1^ FW; **A**), SOD activity (Unit g^−1^ FW; **C**) and MDA content (μmol g^−1^ FW; **E**) in the wheat CRP lines, at various time intervals, upon spot blotch infection were analyzed. A strong negative correlation (*p* ≤ 0.05) of AUDPC to spot blotch-induced H_2_O_2_ **(B)** and SOD **(D)** contents was observed, whereas MDA **(F)** was positively correlated to disease progression. r is the correlation coefficient from Pearson correlation analysis. Susceptible genotypes are marked in red color whereas resistant genotypes are labeled in black.

To determine the basis of differential response of wheat genotypes to spot blotch infection, we measured several signaling components involved in disease resistance. Since ROS play an important role in plant defense against pathogens (Liu et al., 2010), a time course study was performed to analyze H_2_O_2_ accumulation during spot blotch infection in wheat genotypes (to evaluate the ROS activity). For the resistant genotypes, enhanced H_2_O_2_ accumulation was observed as early as 12 hpi (Figure 2A and Supplementary Table 5). Amongst the resistant genotypes, CRP21 showed highest H_2_O_2_ accumulation after 12 hpi (2.4-fold), whereas CRP41 had the lowest H_2_O_2_ accumulation (Figure 2A and Supplementary Table 5). All susceptible genotypes failed to accumulate H_2_O_2_ over the time course of infection. Except CIANO T79 and CRP55, all the susceptible lines clustered together in a single group (Figure 2A). Further, a negative correlation between H_2_O_2_ accumulation and AUDPC of 55 CRP lines was recorded (*r* = −0.60; *P* ≤ 0.05; Figure 2B), indicating that H_2_O_2_ elicitation may be linked to elicitation of defenses against spot blotch infection.

SOD is an antioxidant enzyme that maintains the steady state level of ROS in the plant cells under various stresses. The level of SOD was almost similar in resistant as well as susceptible genotypes before infection (0 hpi), whereas, the activity was elevated in resistant genotypes at 24 hpi, which further increased at 48 and 72 hpi (Figure 2C and Supplementary Table 5). In contrast, susceptible genotypes failed to elicit significant changes in the SOD level within 24 hpi. Although, marginal increase in SOD levels was noted at 72 hpi in susceptible genotypes, these levels were significantly lower than in the resistant lines (Figure 2C and Supplementary Table 5). Overall, SOD level showed a negative correlation to AUDPC (*r* = −0.45; *P* ≤ 0.05; Figure 2D).

Malondialdehyde (MDA), a secondary product of lipid peroxidation, is a commonly used marker of cell membrane damage, and its levels usually increase in response to biotrophic infection (Torres et al., 2006). When we examined MDA content during the time course of spot blotch infection, no significant differences were observed across the genotypes at 0 hpi (Figure 2E and Supplementary Table 5). The MDA levels were increased at 24 hpi; moreover, the levels were high throughout the time course of infection in susceptible genotypes as compared to resistant lines (Figure 2E and Supplementary Table 5). CRP5, a susceptible genotype, showed highest MDA content (0.945 μmoles/g FW) at 72 hpi, followed by CIANO T79 (0.823 μmoles/g FW). All the susceptible genotypes, except Sonalika and CRP39, formed a single cluster (Figure 2E). A strong positive correlation of MDA content and AUDPC was observed (*r* = 0.74; *P* ≤ 0.05; Figure 2F).

### Pathogen infection differentially increases SA and phenolics level in wheat genotypes resistant to spot blotch disease progression

**Figure 3:**
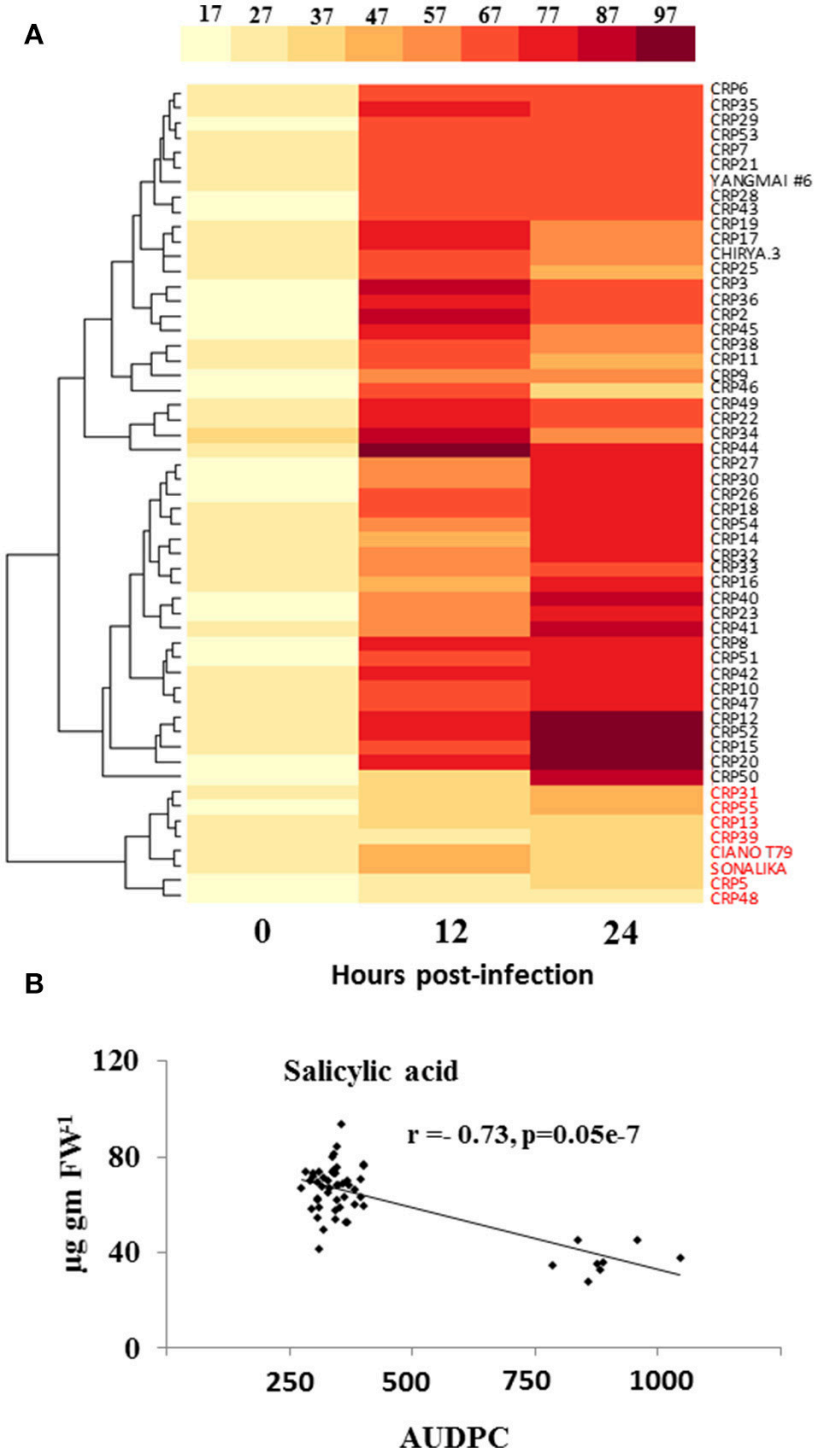
Elicitation of salicylic acid (SA) is strongly related to resistance of wheat against spot blotch. **(A)** Variation in accumulation of SA (μg g^−1^ FW) in 55 wheat genotypes before (0 hpi) and after spot blotch infection is presented with the help of a heatmap. Values on x-axis indicate time. **(B)** An inverse correlation between SA and the AUDPC of 55 CRP genotypes is shown. Red colored labels are susceptible genotypes.

We determined the natural variation in pathogen-induced SA accumulation in wheat and its association to resistance. Our results indicate that the elicitation of spot blotch-induced SA is directly associated with resistance. At 0 hpi, no significant differences were observed for SA levels across 55 wheat genotypes (Figure 3A and Supplementary Table 6). However, at 12 and 24 hpi, the resistant lines displayed an increase in the SA levels (49.62–93.79 μg/g fresh weight; Figure 3A and Supplementary Table 6). CRP45 showed maximum elicitation of 4.5-fold after spot blotch infection at 12 hpi, which remained high at 24 hpi (as compared to time 0). All the susceptible genotypes, including CIANO T79 and Sonalika, did not show significant increase in accumulation of SA after spot blotch infection and formed a single cluster (Figure 3A). The resistant genotypes were grouped in two clusters, with their maximum SA contents at 12 and 24 hpi, respectively (Figure 3A). SA displayed a strong negative correlation of *r* = −0.73 (*P* ≤ 0.05) with AUDPC (i.e., susceptibility; Figure 3B). It may be hypothesized that the failure of eliciting SA-dependent defense signaling may result in increased disease progression (susceptibility of genotypes).

**Figure 4:**
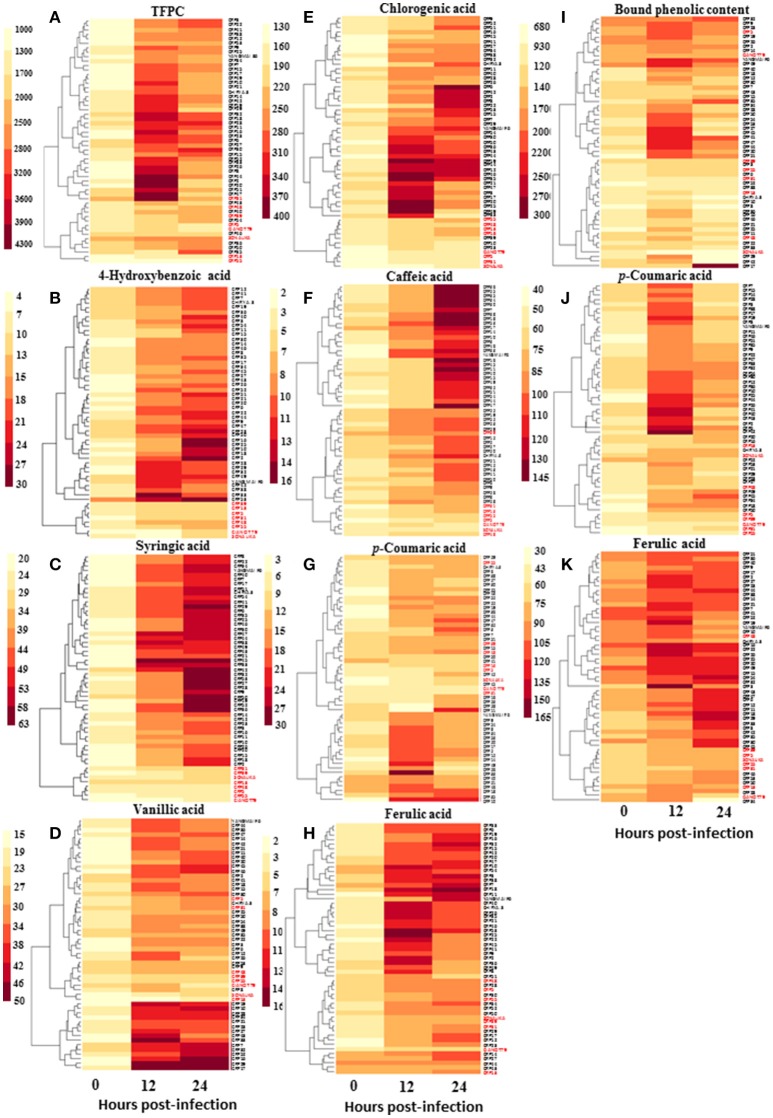
Variation in accumulation of phenolic acids (μg g^−1^ FW) in wheat CRP genotypes during the process of infection of spot blotch. Heat maps show accumulation patterns of **(A)** total free phenolic content, **(B)** 4-hydroxybenzoic acid, **(C)** syringic acid, **(D)** vanillic acid, **(E)** chlorogenic acid, **(F)** caffeic acid, **(G)** *p*-coumaric acid, **(H)** ferulic acid, as well as **(I)** total bound phenolic content, **(J)** *p*-coumaric acid, and **(K)** ferulic acid. In red are the names of susceptible genotypes.

Since phenolic acids and their derivatives are known to play multiple roles in plant pathogen interaction, and that their elicitation may be regulated by SA, we measured spot blotch-induced phenolic acid accumulation in 55 genotypes. A clear increase in accumulation of phenolic acids after *B. sorokiniana* infection was recorded in resistant genotypes than the susceptible genotypes at 12 and 24 hpi (Figure 4 and Supplementary Table 7). Resistant and susceptible genotypes formed two distinct clusters, each, for 4-hydroxybenzoic acid and syringic acid.

**Figure 5:**
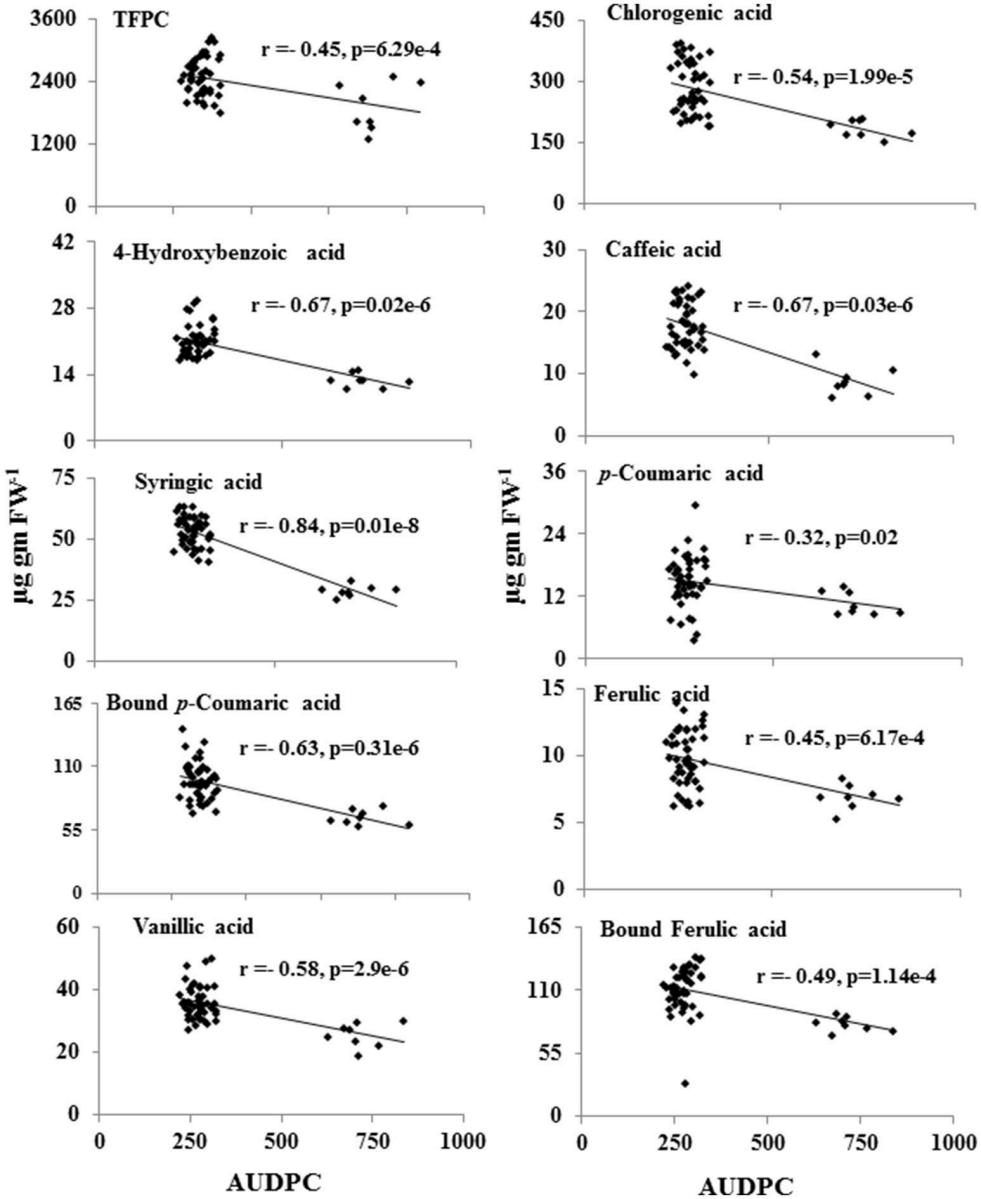
Inverse-correlation between metabolite levels of phenolic acids and the AUDPC of 55 CRP genotypes. Levels of metabolites showing significant correlation (*p* ≤ 0.05) was plotted against AUDPC during spot-blotch infection. r (correlation coefficient) and *p*-values are from Pearson correlation analysis.

Further statistical analysis showed strong inverse correlation between pathogen-induced content of phenolic compounds and the AUDPC (Figure 5). Most strong negative correlation was observed for syringic acid (*r* = −0.84; *P* ≤ 0.05), followed by 4-hydroxybenzoic acid (*r* = −0.67; *P* ≤ 0.05; Figure 5).

### Syringic acid inhibits the growth of *B. sorokiniana* and disease progression

**Figure 6:**
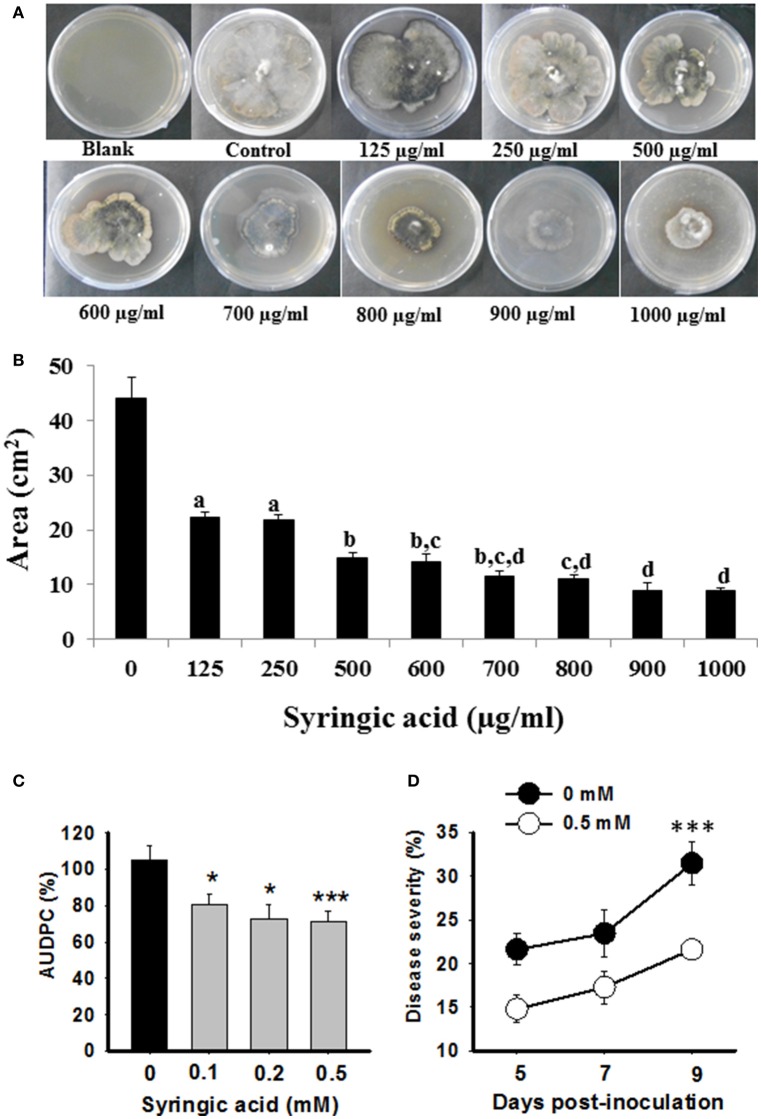
Syringic acid resists *B. sorokiniana* growth and disease progression. Pharmacological evaluations **(A,B)** and exogenous complementation assays **(C,D)** were conducted to determine anti-fungal role of syringic acid. **(A)** Representative pictures of *B. sorokiniana* growing on PDA plates containing different concentrations of syringic acid. **(B)** Area of plate covered by *B. sorokiniana* in presence of syringic acid as compared to the control (no syringic acid) is plotted. **(C,D)** The susceptible (Sonalika) plants were exogenously complemented with various concentrations of syringic acid, and disease severity (right panel; representative data of 0.5 mM vs. 0 mM control Sonalika plants) as well as AUDPC (left panel) were calculated over time course of infection. Values are expressed as mean ± SD for **(C)**. **(D)** Is represented as % of disease severity. One-way ANOVA with Tukey's test was done on absolute values to show significant differences; values with the same letters are not significantly different (*P* ≤ 0.05) from each other, whereas ^*^ and ^***^ indicate significant differences at *P* ≤ 0.05 and *P* ≤ 0.005, respectively.

Due to a strong negative correlation of syringic acid and AUDPC (Figure 5), we hypothesized that it may act as a defensive phytochemical with antimicrobial property. To test direct inhibition of *B. sorokiniana* growth by syringic acid, we performed *in vitro* pharmacological plate assays. The inhibitory effect of syringic acid on fungal growth was evident. A significant inhibition of >40% was observed with as little as 125 μg/ml concentration (Figures 6A,B; one-way ANOVA; Tukey's test; *P* ≤ 0.05), whereas higher concentrations of syringic acid further reduced the growth of *B. sorokiniana* (>80%, compared to control plates, 0 μg/ml; Figures 6A,B; one-way ANOVA; Tukey's test; *P* ≤ 0.05).

To further validate the role of syringic acid in plant defense against spot blotch disease, we performed complementation assays. Sonalika plants, which are highly susceptible to spot blotch and fail to elicit any significant changes in syringic acid after *B. sorokiniana* attack (Figure 4), were subjected to foliar application of 0.1, 0.2, and 0.5 mM syringic acid concentrations. Sonalika plants, exogenously complemented with syringic acid, had lower disease severity as well as AUDPC than the Sonalika control (0 mM) plants treated in identical manner (Figure 6C). Most significant difference was observed at 9 dpi where 0.5 mM syringic acid treated wheat had 31.4% reduced spot blotch severity than untreated plants (Figure 6D). Thus, the strong negative correlation of syringic acid to disease progression, the *in-vitro* growth inhibition assays and exogenous complementation assays, together, show that syringic acid contributes to spot blotch resistance in wheat by, for example, possibly acting as an antifungal compound, whose accumulation is enhanced after spot blotch infection.

## Discussion

We tested the performance of 968 genotypes across five environments in three countries of South-Asia so that we could identify new sources of resistance and understand metabolite basis of variability in resistance against spot blotch in wheat. Our investigation identifies most stable spot blotch resistant genotypes across the environments in South-Asia. Such novel sources of resistance may help in negating effects of fluctuations of selection pressure due to fluctuations in environments from that of natural variation in the genotypes. Although the neutral theory speculates limited effect of genetic polymorphism on fitness (Darwin, 1865; Kerwin et al., 2015), yet many ecologically important traits affect fitness as a consequence of presence of phenotypic variation (Kerwin et al., 2015; Li et al., 2015). We have tested this paradox and conclude that variability in accumulation of signaling and defense metabolites indeed may have fitness consequences in wheat during spot blotch resistance in the field conditions. Our study also creates a link between pathogen-induced expressions in metabolites, as a trait, to the variation in agro-physiological parameters of plant performance under field conditions (Supplementary Table 1). High levels of variations have mostly been studied in model species, such as of *Arabidopsis* and *Nicotiana*, to evaluate defense-metabolism-modulated field fitness (Kerwin et al., 2015; Li et al., 2015), but hardly in wheat. The next step will be to identify the genomic loci that may control such variations in traits contributing to resistance in wheat.

The complex polygenic nature of resistance is a major limiting factor in achieving high and durable levels of resistance against the spot blotch pathogen in wheat. The inclusion of diverse germplasm with broad genetic variability in a breeding program might reveal epistatic interactions controlling quantitative traits. These would also promote recombination events to enhance the chances of getting progenies with improved performance in subsequent generations (Mackay, 2014). Our investigation of natural variation indeed uncovered significant genetic variation for spot blotch resistance amongst 968 wheat genotypes.

A quantitative scoring of disease progression showed remarkable differences between genotypes, suggesting that the mechanisms of plant response are differentially regulated in both the groups (resistant vs. susceptible) of genotypes. This is supported by patterns displayed by components of defense signaling (such as ROS, MDA, SOD, SA, and phenolics such as syringic acid) that mostly accumulate differently in resistant and susceptible genotypes after spot blotch infection. Also, strong negative correlations between defense components and spot blotch progression implied that these signaling pathways are important regulators in plant resistance against fungal infection in wheat. It is clear that defense against spot blotch in wheat is inducible in a signal-dependent manner, and thus raises the possibility of its engineering into susceptible genotypes for durable resistance. Elicitation of SA-dependent signaling appears to be an essentially central component of inducible defense response of wheat against the invading spot blotch pathogen. Earlier reports on natural variation for spot blotch resistance in wheat have largely been focused on determining plant fitness parameters, such as morpho-metric traits associated with disease (Duveiller et al., 2005; Rosyara et al., 2009).

ROS accumulation is considered as one of the earliest defense response after immediate penetration of a pathogen into the host cell (Kadota et al., 2015). ROS are shown to regulate plant defense response following successful recognition of pathogen (Torres et al., 2006; Shetty et al., 2007), whereas a higher concentration of ROS may be toxic to the invading pathogen (Shetty et al., 2003; Camejo et al., 2016). During spot blotch attack, high ROS accumulation was observed in early hours of infection, which may inhibit the pathogen growth as well as induce downstream signaling to activate plant defenses. Consequently, during the later stages of infection of resistant genotypes, ROS levels are reduced while SOD levels are simultaneously increased. It is plausible that fine-tuning between ROS and SOD may help to protect the plants from oxidative damage and may help to confer resistance to wheat against spot blotch. Similar observations are reported previously where H_2_O_2_ and antioxidant enzyme levels correlated positively with plant growth but negatively with the growth of a virulent pathogen (Govrin and Levine, 2002; Mohapatra et al., 2016). On the other hand, MDA, an end product of lipid peroxidation, is often considered as a parameter to evaluate disintegration of cell membrane and DNA damage to plant cells in response to biotic stresses. The high level of MDA following pathogen infection indicates serious injuries to the plant tissue due to oxidative burst (Torres et al., 2006), which corroborates with the susceptibility of the wheat genotypes in our studies. Lower levels of MDA in resistant genotypes after infection indicates that such genotypes may have encountered less oxidative damage as compared to the susceptible genotypes while they defended against the spot blotch infection.

A strong inverse correlation between *Bipolaris*-induced SA accumulation and disease progression was observed. SA plays an important role in the activation of several defense responses against biotrophic pathogens (Vlot et al., 2009). Higher accumulation of SA in the host plant frequently associates with higher disease resistance (Dempsey et al., 1999) and the plants that fail to accumulate active SA are more susceptible to the attack of virulent and avirulent pathogens. In agreement, spot blotch-resistant wheat genotype specifically elicit the change in the SA levels after pathogen infection which, in turn, reprograms the expression of several defense associated genes, ultimately conferring resistance (Sahu et al., 2016b). In contrast, susceptible genotypes failed to display a similar response, indicating that spot blotch induced SA accumulation is an important event to regulate spot blotch resistance in wheat.

Correspondingly, phenolic compounds were elicited after spot blotch infection and their level was significantly high in resistant genotypes than susceptible. Similar changes in overall phenolics content upon spot blotch infection were recorded for resistant recombinant inbred lines (RILs) of wheat (Eisa et al., 2013; Sahu et al., 2016b), which further supported their role in spot blotch resistance. Interestingly, syringic acid not only inhibited the growth of spot blotch pathogens *in vitro*, its application to the susceptible (Sonalika) plants complemented resistance and significantly reduced the disease (Figure 6). These results indicate that syringic acid may act as an active defense by exhibiting antifungal activity against *B. sorokiniana*. Indeed, the increased levels of syringic acid in other disease-inoculated plants have also been shown to inhibit the pathogen growth (Chong et al., 2009; Sánchez-Maldonado et al., 2011; Alves et al., 2013; Shi et al., 2016). Similarly, accumulation of syringic acid was found to be fungitoxic to the growth of *G. boninense* of oil palm (Chong et al., 2009, 2012) and *Didymella* fungus of raspberry (Kozlowska and Krzywanski, 1994). Here, we demonstrate that spot blotch induced syringic acid is strongly negatively related to disease progression and could directly inhibit the pathogen growth. Since syringic acid is fungitoxic in nature (Chong et al., 2009, 2012), it is plausible that reduced spot blotch infection is due to such antifungal activity. These observations suggest that phenolics, such as syringic acid, may be one of the potential biochemical traits for utilization in breeding resistance in wheat against spot blotch.

Overall, our previous observations and the data presented here show that SA and syringic acid are important components of spot blotch resistance (Sahu et al., 2016b; reference herein). However, their exact roles need further investigation. SA and syringic acid share structural similarity as both are the derivatives of C6C1 phenolic compounds. SA is the direct product of benzoic acid whereas syringic acid may be produced through p-hydroxybenzoic acid and vanillic acid. Due to the structural similarity between SA and syringic acid, it is plausible that, following pathogen infection, SA may convert into syringic acid or *vice versa*. As a signaling molecule, SA may elicit defense response by increased accumulation of syringic acid that confers resistance in wheat. Alternatively, syringic acid may also contribute to increased SA accumulation following pathogen infection, thus helping SA in recruitment of other factors such as PR-proteins (Sahu et al., 2016b). Furthermore, phenolic compounds may possibly be involved at later stages of the defense responses such as in preventing pathogen multiplication inside the host, and that their level may be regulated by SA. Elucidating the exact role of these defense-signaling components would be interesting to better understand the spot blotch resistance in wheat.

The next step of investigation, on one hand, would involve cloning and characterization of genes that may participate in spot blotch-induced synthesis of syringic acid in wheat, and on the other hand, to determine the molecular regulatory components of defense signaling to gain further insights into mechanistic details of resistance process. As rightly hypothesized by Soltis and Kliebenstein (2015), correlating variations in abundances of metabolites to variation in resistance across large number of genotypes provided the much needed information on relevant metabolomic networks that are essential for spot blotch resistance. Thus, our study would facilitate undertaking genomics-guided, loci specific investigations for future breeding programs in wheat. Intriguingly, the information generated here may be directly used to rationally design disease management strategies for wheat cultivation in the areas that are hot-spots for spot blotch.

## Author contributions

VM, RC, PS, AJ, SP designed the study. SN, VM, RC, conducted field studies with help from SS and SP. SS, RS, SN, VM, RC, analyzed various data with conceptual help from SP and AJ. RS performed HPLC runs, SN, VM, RC performed measurements related to redox system. PS, AJ, RC, and SP provided resource and coordinated study. SS coordinated revisions with the help from all the authors. SS and SP wrote the MS with contribution of all other authors. All authors read and approved the MS.

### Conflict of interest statement

The authors declare that the research was conducted in the absence of any commercial or financial relationships that could be construed as a potential conflict of interest.
