# Supplementary material for: Natural Variation in Elicitation of Defense-Signaling Associates to Field Resistance Against the Spot Blotch Disease in Bread Wheat (Triticum aestivum L.)
Source: Front Plant Sci. 2018 May 16;9:636. doi: 10.3389/fpls.2018.00636 (PMC5964214; doi:10.3389/fpls.2018.00636)
Supplement: Supplementary file 2 [file Table_2.PDF]

Supplementary Table 2: Statistical analysis of performance of genotypes tested at five geographical locations in 2012-13 and 2013-14.

Supplementary Table 2. 1: Analysis of variance for Environment, Genotype, AUDPC, Days to Heading and TKW of 484 wheat genotypes at all the five locations in 2012-13

**Source of Variance**	**D.F.**	**Mean sum of square**		
		**DH**	**AUDPC**	TKW
Environment	4	68235165.8^{HS}	56490.98^{HS}	13538516.25^{HS}
Block	21	8227.4	10.79	3331.77
Entry	483	54917^{HS}	62.87^{HS}	6528.15^{HS}
Replication	1	94620^{HS}	26.25	2646.22
Environment*Entry	1932	15863.1^{HS}	13.57^{HS}	3816.11
Block*Entry	441	9031.3	7.42	3795.39

^{HS} $p \leq 0.0001$; DH: Days to Heading, AUDPC: Area Under Disease Progress Curve, TKW: Thousand Kernel Weight

Supplementary Table 2.2: Analysis of variance for Environment, Genotype, Days to Heading, AUDPC, TKW of 484 wheat genotypes at all the five locations in 2013-2014.

**Source of variation**	**DF**	**Mean sum of square**		
		**DH**	**AUDPC**	**TKW**
Environment	4	28214750.1^{HS}	10457.77^{HS}	16211991.52^{HS}
Block	21	10836.9	129.21^{HS}	13310.76^{HS}
Entry	483	65591.3^{HS}	42.01^{HS}	5435.1^{HS}
REP	1	495.3	2.62	8848.1
Env*Entry	1932	27312.3^{HS}	8.92	2501.45
Block*Entry	441	10648.7	39.07^{HS}	5392.51^{HS}

^{HS} $p \leq 0.0001$; DH: Days to Heading, AUDPC: Area Under Disease Progress Curve, TKW: Thousand Kernel Weight

Supplementary Table 2.3: Genotypes, selected from the field screening of 2012-13, were ranked on the basis of disease progress curve (AUDPC). Genotypic rankings were calculated by T test (P<0.05).

**Entry No.**	**BHU**	**UBKV**	**BARI**	**RAU**	**NWPR**	**No. of centers showing resistance**	**Ranking**
192	242.22	144.45	49	583.34	656.79	5	A
335	211.14	215.75	96.5	699.99	587.65	5	A
337	193.89	197.23	36.5	650.03	587.65	5	A
89	238.71	195.68	84	666.66		4	B
228		184.26	65	616.67	587.65	4	B
236	239.14	227.47	79	616.67		4	B
294	177.66	187.04	27.5		518.52	4	B
297		184.26	30	583.34	656.79	4	B
346		228.4	71.5	516.65	587.65	4	B

390	302.9	199.39	30	616.67		4	B
483	326.05		41.5	499.98	587.65	4	B
48	262.04		96.5	550.01		3	C
65	202.53		46.5	616.67		3	C
68		184.57	96.5	716.69		3	C
79		227.47	60	616.67		3	C
93	351.92		60.5	616.67		3	C
98			136.5	633.33	656.79	3	C
99		184.26	49	499.98		3	C
153	213.03		60		587.65	3	C
291	345.74		43	533.31		3	C
293		113.89	30		587.65	3	C
301		194.14	71.5	666.66		3	C
321			105.5	633.33	656.79	3	C
336	344.94		104	566.67		3	C
356			67.5	550.01	587.65	3	C
406		221.3	66.5	699.99		3	C
417		204.01	129	600		3	C
419	352.48		53	716.69		3	C
426	223.77		43	583.34		3	C
432	213.02	215.13	110.5			3	C
443	298.03		134	533.31		3	C
Sonalika	830.08	361.11	750	950.01	1093.23		
CINAO T 79	762.72	419.14	556.5	816.01	1002.23		
Max -Min	813.96-177.1	549.39-113.89	750-27.5	950.01-499.98	1093.23-518.52		
**LSD (0.05)**	**176.63**	**115.46**	**143.28**	**291.23**	**172.83**		

Supplementary Table 2.4: Genotypes selected from the screening of 2013-14 were ranked on the basis of disease progress curve (AUDPC). Genotypic rankings were calculated by T test (P<0.05).

**Entry No.**	**BHU**	**UBKV**	**BARI**	**RAU**	**NWPR**	**No. of centers showing resistance**	**Ranking**
364	151.24	444.08	358.33	188	333.77	5	A
473	185.81		383.33	186.5	381.17	4	B
384	194.45		372.53	200.5	416.67	4	B
229	207.41	431.09		194	439.81	4	B
463	155.56	433.56		186.5		3	C
214	168.52			231	330.25	3	C
272	168.52		354.01		466.05	3	C
442	195.66		325.93	248.5		3	C
244	198.77		372.53	188		3	C

457	198.77			211	472.22	3	C
471	203.09		382.1		466.05	3	C
94	207.41	436.91	372.53			3	C
451	207.41			219	466.05	3	C
213	224.69			182	430.56	3	C
40			375.93	205	452.16	3	C
Sonalika	662.96	1132.42	1048.46	719.5	563.27		
CINAO T 79	552.47	750.85	590.43	608	584.88		
Max - Min	662.96-151.24	1146.66-219.88	1048.46-325.93	719.50-182.00	699.07-330.25		
**LSD (0.05)**	**75.6**	**229.35**	**68.0919**	**69.258**	**148.48**		
