# Supplementary material for: Natural Variation in Elicitation of Defense-Signaling Associates to Field Resistance Against the Spot Blotch Disease in Bread Wheat (Triticum aestivum L.)
Source: Front Plant Sci. 2018 May 16;9:636. doi: 10.3389/fpls.2018.00636 (PMC5964214; doi:10.3389/fpls.2018.00636)
Supplement: Supplementary file 8 [file Presentation_1.PDF]

Supplementary material

Natural variation in elicitation of defense-signaling associates to field resistance against the spot blotch disease in bread wheat (*Triticum aestivum* L.)

Sandeep Sharma[1‡], Ranabir Sahu[2‡], Sudhir Navathe[3], Vinod K. Mishra[3], Ramesh Chand[3], Pawan K. Singh[4], Arun K. Joshi[5], Shree P. Pandey[2*]

***Corresponding author**

Shree P. Pandey (shreeppandey@gmail.com)

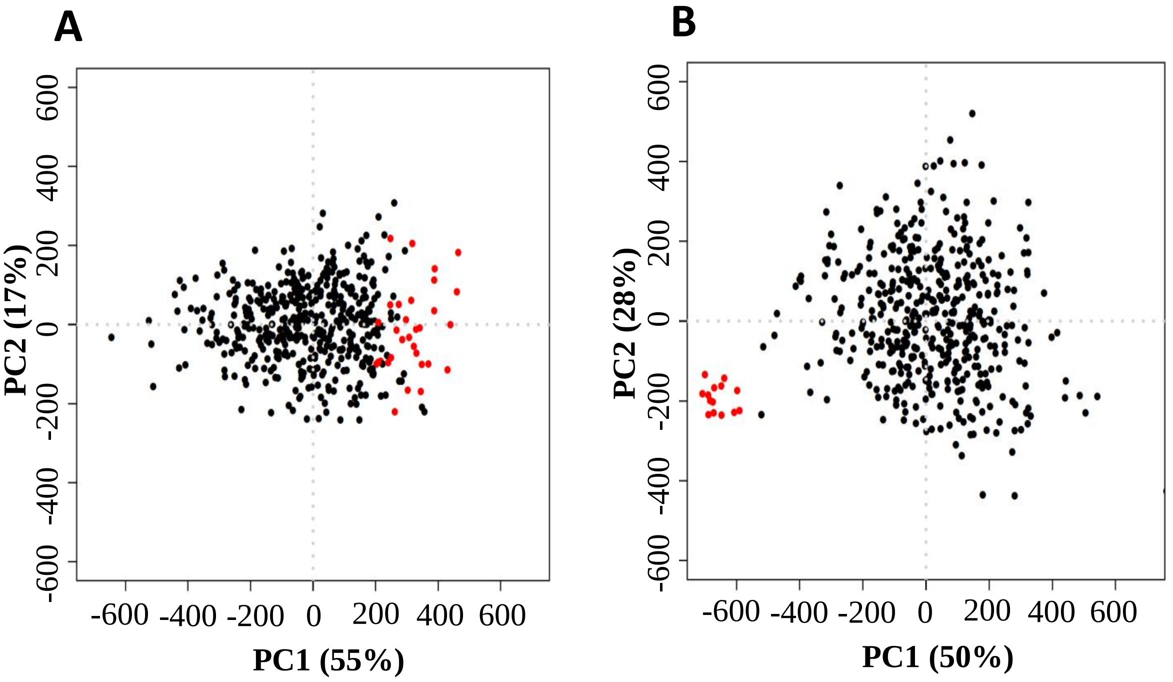

Supplementary Figure 1: Principal component analysis was conducted to discriminate genotypes resistant to spot blotch than the rest. PC1 and PC2 represents the percent (%) variance of the AUDPC over all five geographical locations in the Year 2012-13 (A) and the year 2013-14 (B).

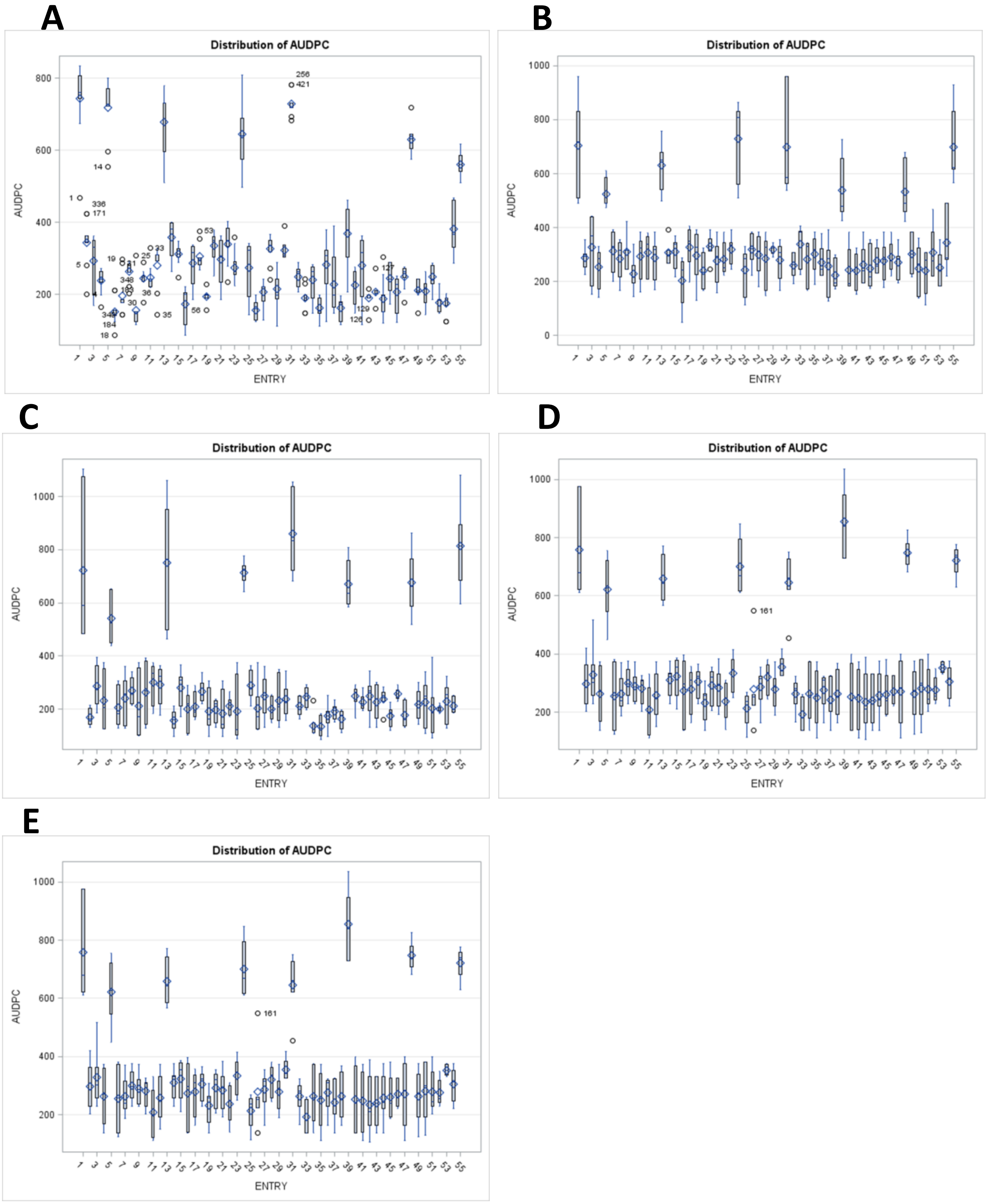

Supplementary Figure 2: The distribution of AUDPC of 55 wheat genotypes at five field locations (A. BHU, B. BARI, C. UBKV, D. RAU, and E. NWPR) are plotted. The susceptible genotypes (CRP1, CRP5, CRP13, CRP24, CRP31, CRP39, CRP48 and CRP55) have significantly higher AUDPC and are separated from the resistant (details of statistics are given in Supplemental Table 3).
